# Red (660 nm) or near-infrared (810 nm) photobiomodulation stimulates, while blue (415 nm), green (540 nm) light inhibits proliferation in human adipose-derived stem cells

**DOI:** 10.1038/s41598-017-07525-w

**Published:** 2017-08-10

**Authors:** Yuguang Wang, Ying-Ying Huang, Yong Wang, Peijun Lyu, Michael R. Hamblin

**Affiliations:** 1 Center of Digital Dentistry, Peking University School and Hospital of Stomatology, Beijing, China; 2 National Engineering Laboratory for Digital and Material Technology of Stomatology, Beijing, China; 3 Wellman Center for Photomedicine, Massachusetts General Hospital, Boston, MA 02114 USA; 4 Department of Dermatology, Harvard Medical School, Boston, MA 02115 USA; 5 Harvard-MIT Division of Health Sciences and Technology, Cambridge, MA 02139 USA

## Abstract

We previously showed that blue (415 nm) and green (540 nm) wavelengths were more effective in stimulating osteoblast differentiation of human adipose-derived stem cells (hASC), compared to red (660 nm) and near-infrared (NIR, 810 nm). Intracellular calcium was higher after blue/green, and could be inhibited by the ion channel blocker, capsazepine. In the present study we asked what was the effect of these four wavelengths on proliferation of the hASC? When cultured in proliferation medium there was a clear difference between blue/green which inhibited proliferation and red/NIR which stimulated proliferation, all at 3 J/cm^2^. Blue/green reduced cellular ATP, while red/NIR increased ATP in a biphasic manner. Blue/green produced a bigger increase in intracellular calcium and reactive oxygen species (ROS). Blue/green reduced mitochondrial membrane potential (MMP) and lowered intracellular pH, while red/NIR had the opposite effect. Transient receptor potential vanilloid 1 (TRPV1) ion channel was expressed in hADSC, and the TRPV1 ligand capsaicin (5uM) stimulated proliferation, which could be abrogated by capsazepine. The inhibition of proliferation caused by blue/green could also be abrogated by capsazepine, and by the antioxidant, N-acetylcysteine. The data suggest that blue/green light inhibits proliferation by activating TRPV1, and increasing calcium and ROS.

## Introduction

Adipose-derived stem cells (ASCs) have recently emerged as a popular and versatile tool in the field of regenerative medicine^1^. Previously bone marrow was the most often employed source of stem cells, including mesenchymal stem cells. However in humans, bone marrow aspiration is a relatively painful and invasive procedure. Adipose tissue is easily isolated in the form of fat removed during liposuction procedures^2^. ASCs will undergo proliferation when cultured in proliferation medium, while they will undergo various differentiation pathways when they are cultured in the appropriate medium with addition of a number of lineage-specific factors. For instance they will differentiate into osteoblasts, when cultured in osteogenic medium containing factors such as 25-dihydroxyvitamin D3, glycerophosphate, ascorbic acid, bone morphogenetic protein-2, or valproic acid^3^. Differentiation of ASCs into osteoblasts may have applications to produce cells that could replace autologous bone grafts (which are usually harvested from the iliac crest)^4^. They could overcome limitations of this procedure such as additional morbidity, limited availability, and a requirement for a second surgical procedure^5^.

Photobiomodulation (PBM), formerly known^6^ as low-level laser therapy (LLLT), is a rapidly growing approach to many medical indications requiring reduction of pain and inflammation, and stimulation of healing and tissue regeneration^7^. The term “photobiomodulation” is considered to be more appropriate since PBM can either stimulate or inhibit biological processes depending on the wavelength and energy dose. PBM has traditionally employed red (600–700 nm) and near-infrared (NIR, 780–1100 nm) wavelengths^8^. The primary chromophore for these wavelengths is generally considered to be cytochrome c oxidase, which is unit IV of the mitochondrial respiratory chain^9^. However evidence is mounting that shorter visible wavelengths such as blue and green light can also have PBM effects^10–12^, and these effects may show distinct differences from those generally observed with red/NIR light^13^.

In a previous report^13^ we showed that blue (415 nm) and green (540 nm) wavelengths were more effective in stimulating osteoblast differentiation of human adipose-derived stem cells (hASC) cultured in osteogenic medium, compared to red (660 nm) and near-infrared (NIR, 810 nm) light. Intracellular calcium was higher after 415 nm and 540 nm, and could be inhibited by the ion channel blocker, capsazepine. In the present study we asked what was the effect of these four wavelengths on proliferation of the hASC cultured in proliferation medium?

## Materials and Methods

### Cell culture

Human adipose-derived stem cells (hASCs) were purchased from ScienCell Company (San Diego, CA, USA). Dulbecco’s modified Eagle’s medium (DMEM), 100× penicillin and streptomycin mixture were purchased from Sigma-Aldrich (St. Louis, MO, USA). Fetal bovine serum (FBS) is purchased from Atlanta biologicals (Flowery Branch, GA, USA). All other materials were purchased from Sigma-Aldrich unless otherwise stated. hASCs were cultured in fresh DMEM containing 10% (v/v) FBS, 100 U/mL penicillin G and 100 mg/mL streptomycin at 37 °C in an incubator with an atmosphere consisting of 95% air, 5% CO_2_ and 100% relative humidity. Cells from the fourth to seventh passage were used for the *in vitro* proliferation experiments.

### Irradiation protocol and pharmacological compounds

*hASCs were irradiated by 4 different wavelengths (415, 540, 660, 810* *nm) photobiomodualtion at the dose of 3* *J/cm* ^2^ *in cell proliferation medium (*Table 1 *). Capsaicin (CAP) is an agonist of* transient receptor potential vanilloid 1 (TRPV1) channel. *Capsazepine (CPZ) is* a selective inhibitor of TRPV1 channel. N-Acetyl-L-cysteine (NAC) is commonly used to inhibit ROS (reactive oxygen species). CAP and CPZ were dissolved in DMSO and a final concentration 5 μM was used (final DMSO concentration <0.5%). The final concentration of NAC was 5 mM.

**Table 1:** Light equipment and parameters.

Wavelength	400–430 nm	525–555 nm	660 nm	810 nm
Light Type	LED array	Filtered lamp	Diode laser	Diode laser
Manufacturer	OMNILUX, CA	LumaCare™ Lamp, CA	Arroyo Instruments, LLC, CA, USA	Opto Power Corp., Tucson, AZ, USA
Models	D35PN EL 1600	Model LC-122 Medical	5305 TECSource, 5 A/12 V,4308 LaserSource, 8 A	Model D030-MM-FCTS/B
Mode	CW	CW	CW	CW
Fluence rate (mW/cm^2^)	16	16	16	16
Time of irradiation (s)	188	188	188	188
Fluence (J/cm^2^)	3	3	3	3
Spot size (cm^2^)	4	4	4	4

The fluence rate was adjusted by changing the distance between the laser and the cell culture dish. The cell culture plates were covered with aluminum-foil, spot size was defined by the size of window in the aluminum-foil. CW, continuous-wave.

### Sulforhodamine B colorimetric assay

*The cell proliferation assay was determined using the sulforhodamine B assay (SRB). Briefly*, hASCs were seeded in 96-well plate at a density of 3,000 per well and cultured for 24 h. After laser irradiation, the cells were fixed with 10% (wt/vol) trichloroacetic acid and stained by adding 100 uL of 0.057% (wt/vol) SRB solution to each well. After removing the excess dye by washing with 1% (vol/vol) acetic acid, the cells were dissolved in 10 mnM Tris base solution. A 96-well plate reader (SPECTRA-max M5, Molecular Devices, USA) was used to determine the OD at 510 nm.

### ROS Production

The ROS was measured using CM-H2DCFDA (General Oxidative Stress Indicator), a fluorescent indicator for reactive oxygen species. After light delivery, the cells were incubated with 10 μM of CM-H2DCFDA in PBS solution 30 mins in dark at 37 °C. The cells were washed twice and prepared for flow cytometry or 96-well plate reader.

### Intracellular Calcium Assay

Fluo-4 AM was used to monitor changes of intracellular calcium. Briefly, the cells were pretreated with 1 μM Fluo-4 AM 1 h in dark at 37 °C, and then washed three times with PBS. Light was then applied to the cells and confocal images or flow cytometry followed immediately.

### Intracellular pH Assay

To monitor changes of intracellular PH, BCEF-AM was loaded to hASCs at a final concentration of 50 nM at 37 °C for 30 min. hASCs were washed with live Cell Imaging Solution (LCIS for short, Invitrogen, Darmstadt, Germany) and followed by flow cytometry.

### Mitochondrial Membrane Potential (MMP) Measurement

To determine changes in the mitochondrial membrane potential, tetramethylrhodamine, methyl ester (TMRM) was loaded into the cells at a concentration of 25 nM at 37 °C for 15 min. Cells were then washed with live Cell Imaging Solution (LCIS, Invitrogen, Darmstadt, Germany), A sterile-filtered, 2 M glucose stock solution was added into LCIS to support cell health over longer time periods. Cells were analyzed by confocal microscopy or by flow cytometry.

### ATP Assay

The hASCs were seeded in opaque-walled 96-well white polystyrene plates (Corning Life Sciences, Acton, MA, USA) at relatively low density (2*10^3^ cells/well) and incubated the cells at 37 °C, 5% CO_2_ for 3 days for ATP assay. A value for background luminescence was obtained in control wells containing medium without cells. The medium of each well was adjusted to100 µL and the plate incubated at room temperature for approximately 30 minutes. Addition of 100 µl of CellTiter-Glo Luminescent Cell Viability Assay reagent (Promega Corp, Madison, WI, USA) to the 100 µl of medium present in each well. The contents were agitated for 2 minutes on an orbital shaker to induce cell lysis. The plate was incubated at room temperature for 10 minutes to stabilize the luminescent signal. The luminescence was measured with a SpectraMax M5 Multi-Mode Microplate Reader (Molecular Devices, Sunnyvale, CA, USA).

### Western Blot

hASCs was re-suspended in 2× packed cell volume (PCV) of RIPA lysis buffer (Thermo) supplemented with 1% phosphatase and protease inhibitor cocktail respectively (Sigma, Millipore). The mixture was incubated on a shaker (1 h, 4 °C) and sonicated (2 min twice). Subsequent to centrifugation (14 000 rcf, 10 min, 4 °C), supernatants were collected and protein concentrations were determined using the BCA assay (Bio-Rad).

Protein expression was analyzed using a standard Western protocol (Bio-Rad). As briefly described, protein lysates 10 μg were separated on 4–20% precast polyacrylamide gel (Mini-PROTEAN TGX, Bio-Rad) and transferred onto PVDF membrane (Thermo). Subsequent to blocking with 5% milk in TBST solution, proteins were further detected using antibodies against TRPV1 (1:1000). Anti β-actin antibodies (1:5 000, Cell Signaling) were used for loading control. Visualization of protein bands were developed by chemiluminescence (ECL, Bio-Rad) with exposure to X-ray film (Thermo).

### Statistical Analysis

All assays were performed in triplicate with n = 6/9 for every sample. SPSS 19.0 (SPSS Inc., Chicago, IL, USA) was used to perform Single-Factor ANOVA with a Tukey’s post-hoc test to evaluate the statistical significance of experimental results (P < 0.05). For multiple comparisons, Bonferroni was used in all the experiments. The 2^delta delta Ct method was used in relative gene expression studies.

## Results

### Cell proliferation is increased after PBM with 660 nm and 810 nm, but is decreased after 415 nm and 540 nm

**Figure 1:**
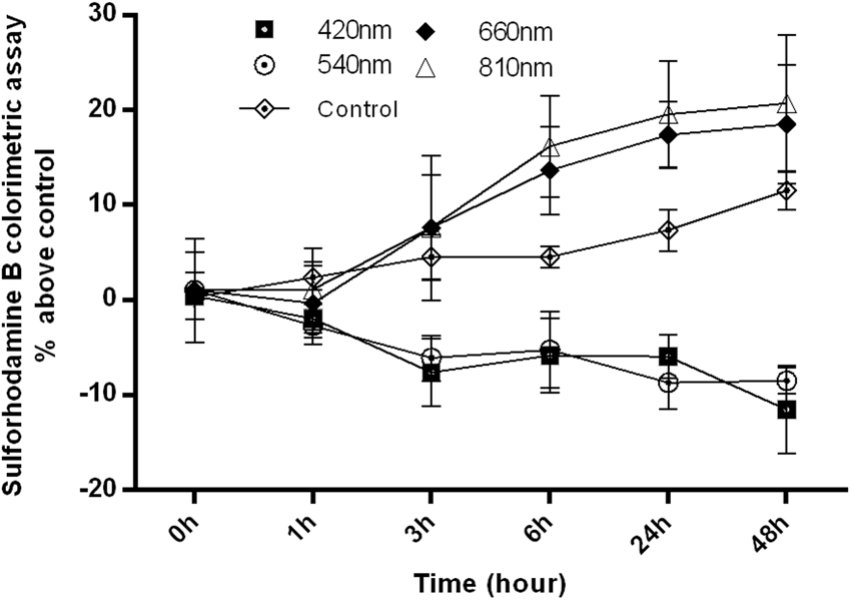
Effect of different wavelength PBM (3 J/cm^2^) and no light (dark) on cell proliferation: 660 nm and 810 nm enhanced the cell proliferation, but 415 nm, 540 nm PBM decreased the proliferation of the cells. Dark led to only modest proliferation. Data represent mean ± SEM (n = 9).

To characterize the effects of different wavelengths of PBM on the proliferation of hASCs, we used the sulforhodamine B assay which is based on the measurement of cellular protein content to determine the cell density. In order to detect the time course of cell proliferation after PBM, we used 4 different wavelengths of light (3 J/cm^2^) to stimulate the cells at 5 time points (48 h, 24 h, 6 h, 3 h, 1 h) before the SRB assay. We found that 3 J/cm^2^ of 810 nm and 660 nm laser promoted hASCs proliferation, but 415 nm and 540 nm wavelengths showed an inhibitory effect on proliferation at the same dose. The time courses are shown in Fig. 1.

### Intracellular ATP levels show a biphasic increase with 660 nm and 810 nm, but a dose dependent decrease with 415 nm and 540 nm

**Figure 2:**
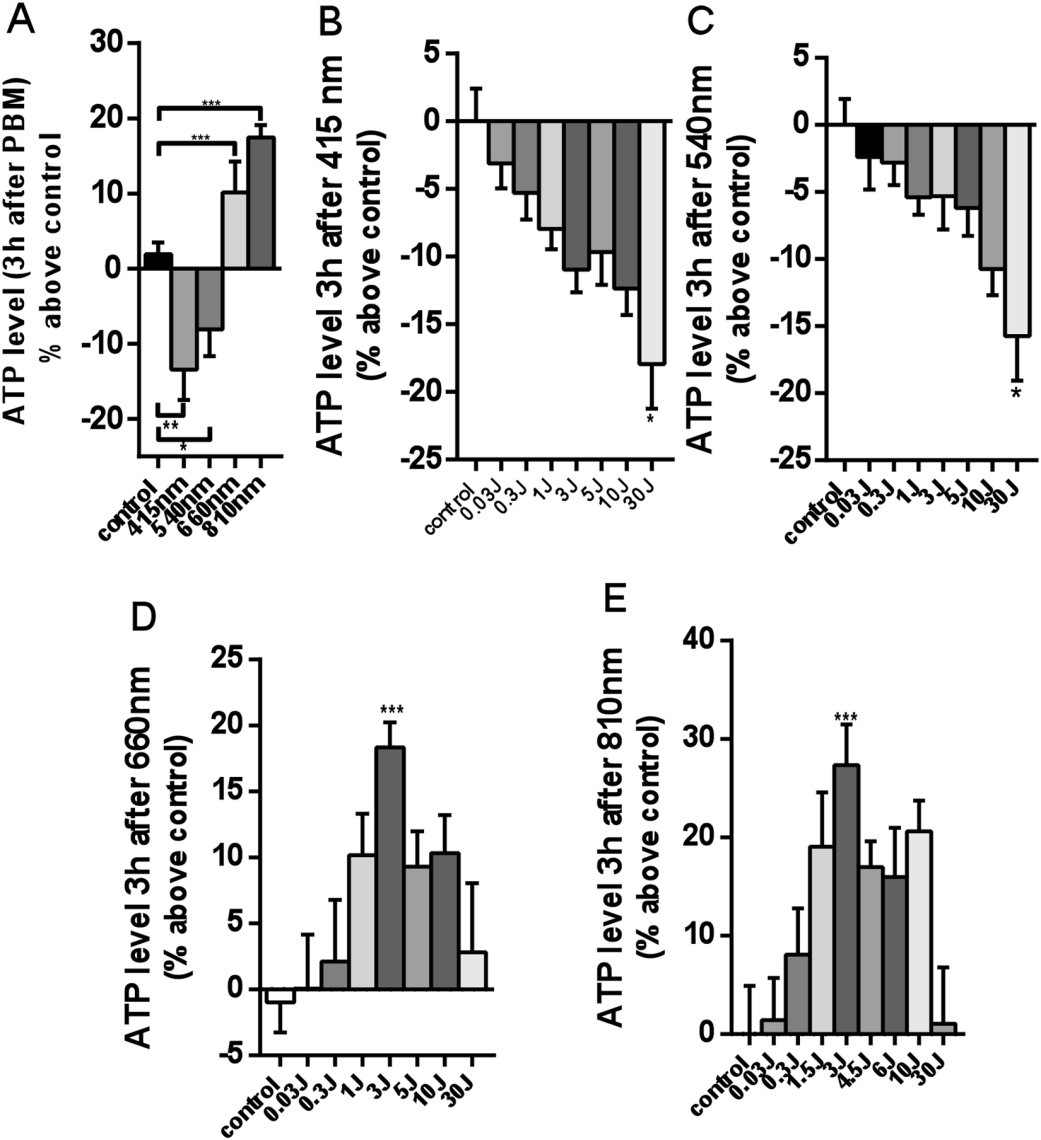
Quantitative analysis of intracellular ATP production level 3 h after PBM. Data represent mean ± SEM. All data are expressed as percentage above control. (n = 9; *P < 0.05, **P < 0.01, ***P < 0.001).

To examine why 3 J/cm^2^ of blue and green PBM inhibited cell proliferation, while red and NIR PBM enhanced cell proliferation, we irradiated the cells with different dosages and measured the intracellular ATP content three hours after irradiation. We found that 3 J/cm^2^ of 660 nm and 810 nm PBM could increase intracellular ATP level by 15–20%, while 3 J/cm^2^ of 415 nm and 540 nm decreased intracellular ATP level in the region of 10%, 3 h after irradiation (Fig. 2A). We also found that 660 nm and 810 nm showed a biphasic dose response, lower doses showed stimulation of ATP, while high doses (30 J/cm^2^) of 660 nm and 810 nm light produced progressively lower ATP increases until background levels were reached at 30 J/cm^2^ (Fig. 2D,E). 660 nm and 810 nm both exhibited a peak dose response 3 h after irradiation at 3 J/cm^2^ (P < 0.001). In contrast, 415 nm and 540 nm, showed a progressively linear reduction in ATP with increasing light doses to a maximum of 30 J/cm^2^, (Fig. 2B,C).

### Effects of 415 nm and 540 nm compared with 660 nm and 810 nm on intracellular calcium, mitochondrial membrane potential (MMP), intracellular ROS and intracellular pH

**Figure 3:**
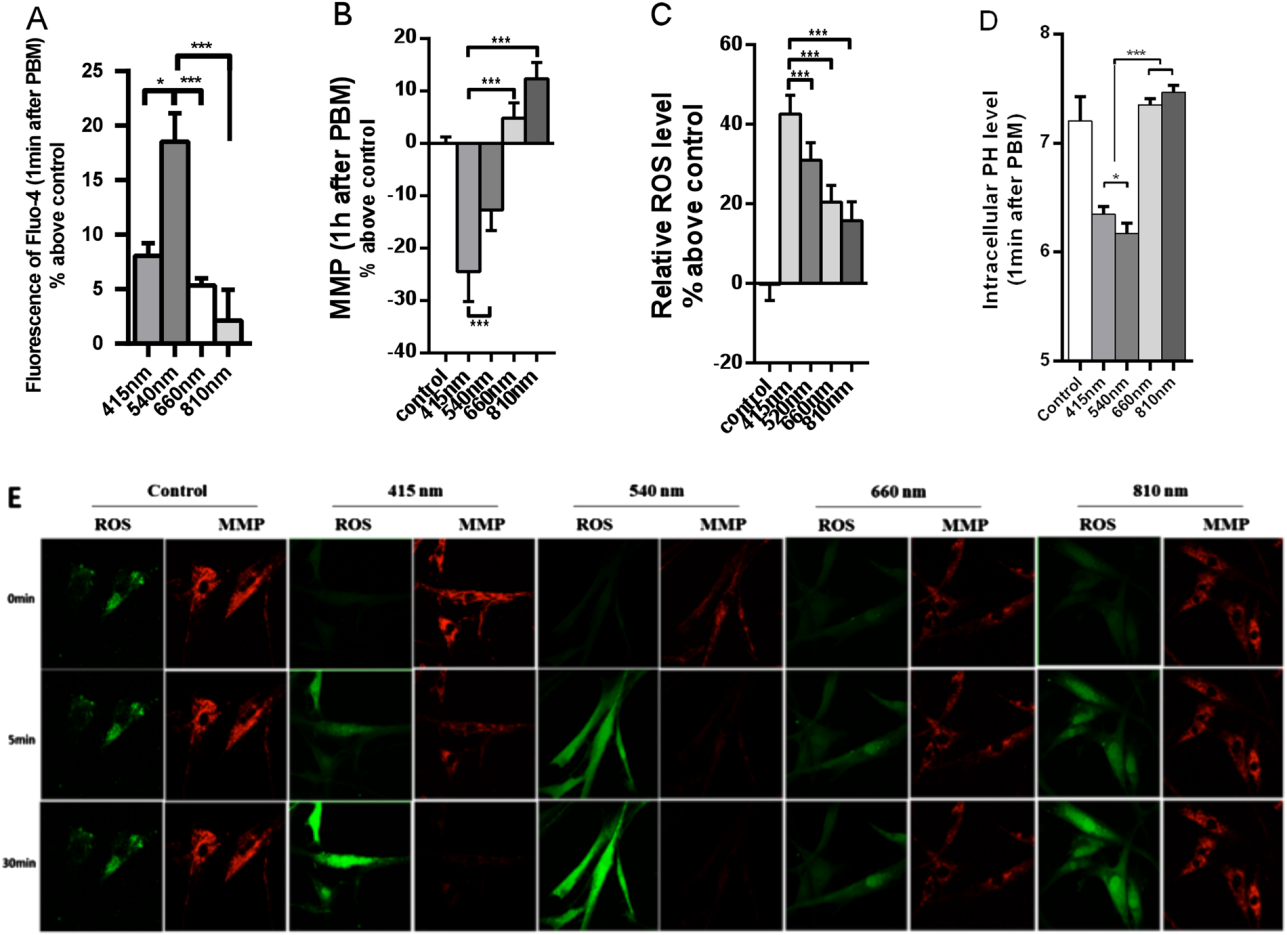
Quantitative evaluation of 4 different wavelengths of PBM (3 J/cm^2^) on intracellular calcium level 1 min after PBM (**A**); MMP 1 h after PBM (**B**); intracellular ROS 1 h after PBM; (**C**) Absolute values of intracellular pH 1 min after PBM (**D**). Data represent mean ± SEM. (n = 9; *P < 0.05, ***P < 0.001) all data using flow cytometry. (**E**) Fluorescent confocal microscopy images of cellular MMP 5 min after PBM: and intracellular ROS 30 min after PBM.

In the following set of experiments, we compared the effects of 4 different wavelengths of PBM all delivered at the sane dose of 3 J/cm^2^ on intracellular calcium (Fig. 3A), MMP (Fig. 3B), intracellular ROS (Fig. 3C), and intracellular pH (Fig. 3D) by flow cytometry. We verified our results using images taken by confocal microscopy (Fig. 3E). Figure 3A shows that 3 J/cm^2^ of 415 nm and 540 nm significantly increased intracellular calcium level 1 min after PBM, and the same dose of 540 nm showed the most pronoinced effect amongst the 4 different wavelengths tested (#P < 0.05, ***P < 0.001). Figure 3B shows 3 J/cm^2^ of 415 nm had the greatest impact on reducing MMP by about 30 percent 1 hour after PBM, while 3 J/cm^2^ of 540 nm PBM also reduced MMP, but not so much as blue light. In contrary red and NIR light increased MMP by 8-15% at a dose of 3 J/cm^2^ (***P < 0.001). Figure 3C shows that all four different wavelengths of PBM increased intracellular ROS level at a dose of 3 J/cm^2^, blue light increased ROS most (about 45%), while green, red and NIR showed progressively less ROS generation (***P < 0.001). Figure 3D shows that both 415 nm and 540 nm PBM reduced the intracellular pH level, with 540 nm having the most effect. 660 nm and 810 nm groups slightly (but significantly) showed increased intracellular pH values (*P < 0.05, ***P < 0.001). In Fig. 3E, images of ROS and MMP taken with the confocal microscope are shown. hASCs were irradiated with four different wavelengths PBM and images were taken 5 and 30 minutes after irradiation showing the same trend as the flow cytometry data in Fig. 3A–D. Blue and green light produced a large amount of ROS, and decreased MMP, while red and NIR produced a small amount of ROS, and increased the MMP level.

### Intracellular Ca2+ ion channel TRPV1 expression can be detected in hASCs, a low concentration of the TRPV1 agonist capsaicin promotes cell proliferation, this effect can be blocked by capsaicin TRP inhibitor

**Figure 4:**
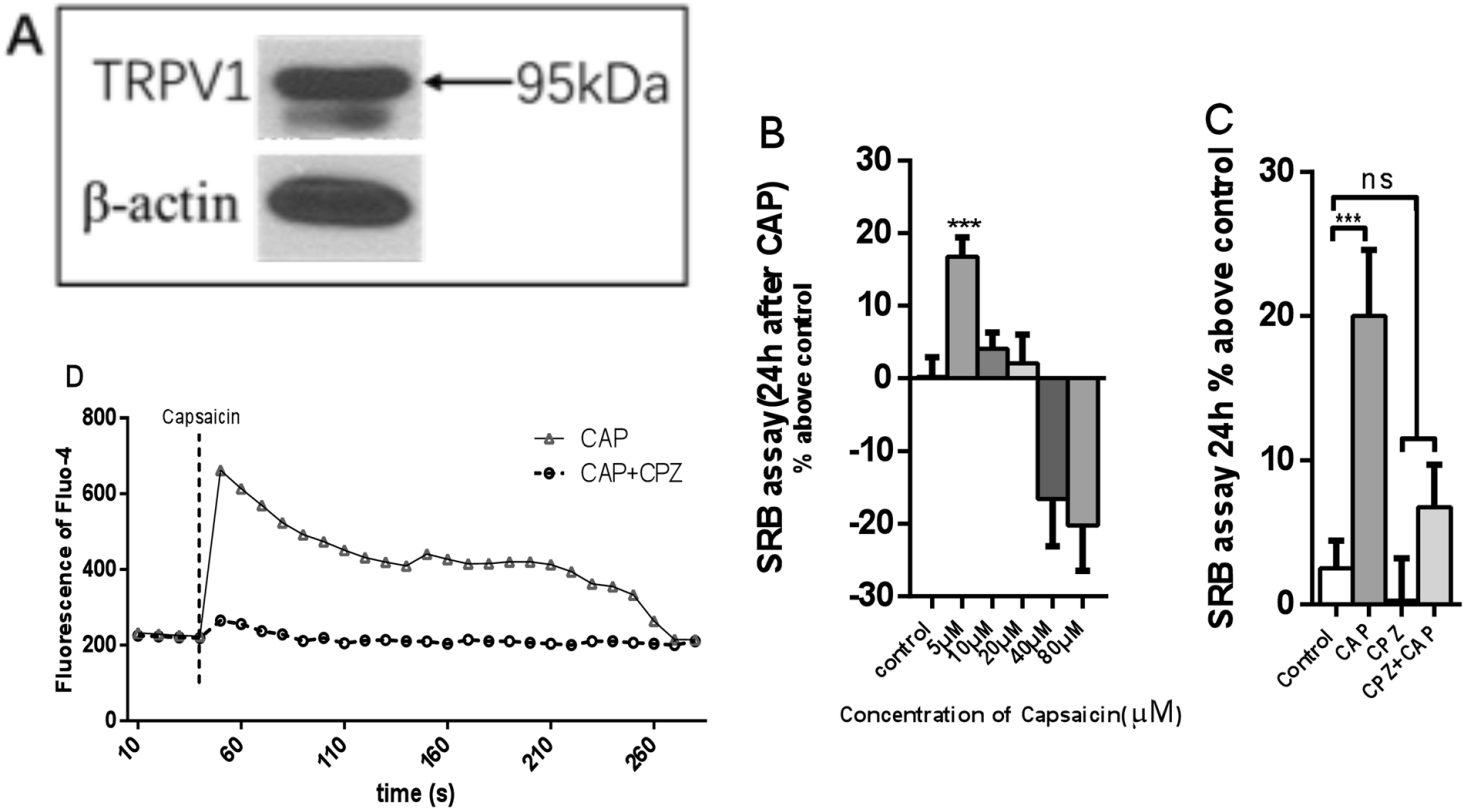
TRPV1 expression and functionality in hASCs. (**A**) Expression of TRPV1 in hASCs. (**B**) Cell proliferation after different concentrations of capsaicin. (**C**) Cell proliferation after capsaicin (CAP) with or without capsazepine (CPZ). (**D**) Time course of intracellular calcium level after CAP and CAP + CPZ. (n = 9, ***P < 0.001)

In order to detect whether the proliferation of cells was related to the increase of intracellular calcium concentration, we detected the expression of TRPV1 calcium channel by western blot. As show in Fig. 4A, hASCs expressed TRPV1 detected by western blot, anti β-actin antibodies were used for loading control. We stimulated the cells with the TRPV1 agonist capsaicin (CAP) in increasing concentrations measured the effects on cell proliferation 24 h later (Fig. 4B). We found that a low concentration of capsaicin (5 μM) in a short-term incubation (incubated with capsaicin for 5 min then washed with PBS), could promote cell proliferation (Fig. 4B) but higher concentrations (10 and 20 μM) and no effect, and even higher concentrations of CAP (40 and 80 μM) could inhibit proliferation. The effect of 5 μM CAP on promoting cell proliferation could be abrogated by 5 μM of the TRPV1 antagonist capsazepine (CPZ) (Fig. 4C). CPZ also blocked the rapid increase (1 min) in intracellular calcium caused by CAP (Fig. 4D).

### The inhibitory effects of 415 nm and 540 nm on cell proliferation could be abrogated by TRPV1 channel blocker (CPZ) and ROS inhibitor (NAC)

**Figure 5:**
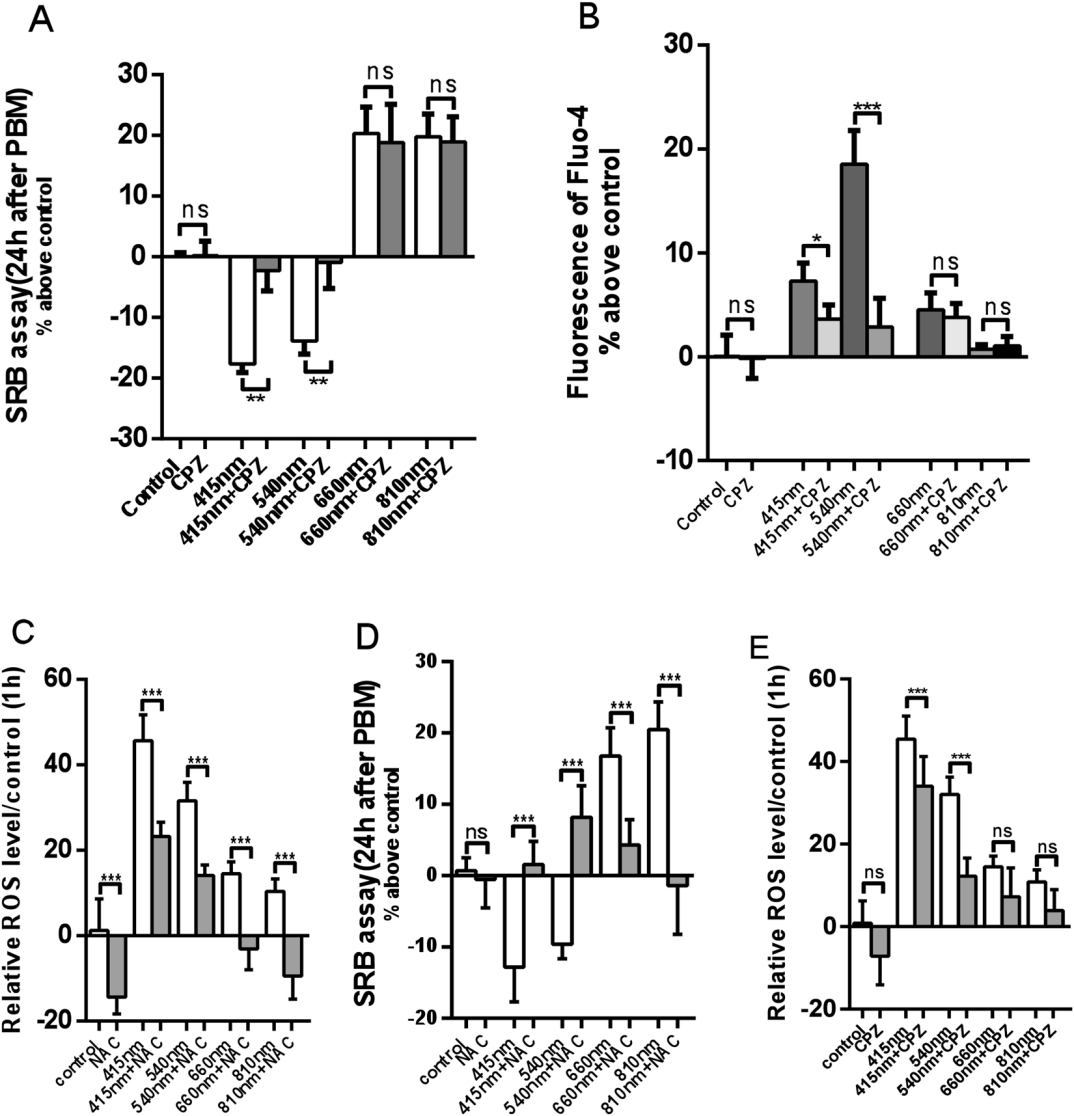
(**A**) Quantitative evaluation of cell proliferation 24 hour after PBM with pretreatment with CPZ. (**B**) Intracellular calcium level 1 min after PBM with pretreatment with CPZ. (**C**) Intracellular ROS level 1 hour after PBM with pretreatment with NAC. (**D**) Quantitative evaluation of cell proliferation 24 hour after PBM with pretreatment with NAC. (**E**) Intracellular ROS level 1 hour after PBM with pretreatment with CPZ. (n = 9; *P < 0.05, **P < 0.01, ***P < 0.001).

In order to investigate whether the inhibitory effects of blue and green light on cell proliferation operated via activation of the TRPV1 calcium channel and/or increased intracellular ROS levels, we pretreated hASCs with 5 μM of CPZ or 5 mM of ROS inhibitor N-acetyl-L-cysteine (NAC) 10 min before PBM. We found that pretreatment by CPZ could significantly abrogate the intracellular calcium elevation caused by blue light and green light (Fig. 5B). While red light and near infrared light had little effect on intracellular Ca2+ level, and there was no significant differences between CPZ pretreatment and non-treatment groups (Fig. 5B). NAC could significantly reduce the intracellular ROS level, and was effective for all experimental groups (Fig. 5C). We also found that low concentrations of CPZ and NAC alone had no effect on cell proliferation after 24 hours, but after CPZ and NAC pretreatment, the inhibitory effects of blue and green light on cell proliferation was abrogated. In contrast, CPZ and NAC pretreatments did not affect the activity of red and infrared light (Fig. 5A,D). To see whether the increase of ROS caused by PBM was connected with the activation of TRPV1 ion channel, we examined the effect of TRPV1 inhibitor CPZ on ROS levels. Figure 5E shows there was a modest (but significant, P < 0.001) reduction (about 25%) produced by CPZ on the ROS triggered by blue light, but a more pronounced reduction (about 60%) on the ROS produced by green light (P < 0.001). There was no significant reduction by addition of CPZ on the already modest levels of ROS produced by red/NIR light.

## Discussion

The present study, in conjunction with our previous study^13^, has found interesting differences between these four wavelengths of PBM. Broadly speaking there appears to be distinct differences between blue and green light on the one hand, and red and NIR light on the other hand. The two studies also highlight interesting differences between differentiation and proliferation programs in hASC. We previously found^13^ that blue/green wavelengths were more effective in triggering osteogenic differentiation (when the hASC were cultured in osteogenic medium, compared with same dose of red/NIR. The involvement of TRP ion channels in this mechanism was implicated by the finding that ion channel inhibitors CPZ and SKF96365 (SKF) abrogated the osteogenic differentiation produced by blue/green light.

The literature reports of the effects of blue light on various cellular processes are highly confusing. Most of the studies however do agree that the overall effect of blue light PBM is to increase the level of cellular ROS. This ROS increase is likely to inhibit proliferation in many cases^14, 15^, and can even produce apoptosis and outrightcytotoxicity if the doses are high enough^16, 17^. However matters are complicated by the fact that the term “blue light” has been used to refer to wavelengths spanning a range of over 100 nm. Violet light (395–425 nm) is known to be antibacterial and the chromophore responsible for this action is thought to be generation of ROS via photoexcitation of metal free porphyrins that have a large Soret band at about 405 nm^18^. Royal blue light (430–470 nm) is also known to generate ROS and in this case the chromophores are thought to be flavins, flavoproteins or cryptochromes^19^. However, it is not clear to what extent cryptochromes are expressed in mammalian cells compared to insects, plants and marine organisms^20^. Blue-green light (475–510 nm) is thought to be primarily absorbed by opsins (OPNs). Opsins are a group of cis-retinal dependent G-protein coupled receptors (the best known of which are the visual pigments (present in the retinal photoreceptors (OPN1 in cones and OPN2 in rods)^21^. However, there are now 5 known members of the mammalian opsin family, all of which have different absorption maxima^22^. OPN3 is called encephalopsin or panopsin and absorbs blue-green light^23^, OPN4 is called melanopsin and has a maximum absorption at 480 nm^24^ while OPN5 is called neuropsin and absorbs 380 nm^25^. Opsins signal via two main pathways depending on the type of G-protein they are coupled with^26, 27^. Those opsins (OPN1, OPN2, OPN3, OPN5) that are coupled with G_o_, G_i,_ G_t_, G_s_ proteins signal via a pathway involving cyclic nucleotides (cAMP and cGMP). On the other hand OPN4 is coupled to Gq and signals via the phospholipase C pathway leading to production of inositol triphosphate and di-acylglycerol. It is known that activation of retinal opsins by blue light can generate ROS, which is responsible for ocular phototoxicity caused by violet and blue light^28^. One of the main downstream signaling pathways activated by opsins, is related to members of the family of transient receptor potential (TRP) cation channels such as TRPV1 (vanilloid sub-family). TRP channels are a superfamily of 28 members identified by amino acid sequence similarities and sub-divided into organized into six subfamilies^29^. TRPV1 (capsaicin receptor) has been shown to be activated by various wavelengths of light, including green, red and NIR^30–33^. The first law of photobiology states that there must be a specific photoacceptor molecule (or chromophore) for any biological effect to be caused by light^34^. At present the leading hypothesis to explain the light activation of TRP channels, is that the photons (especially blue and green photons) are absorbed by light-sensitive proteins in the family of mammalian opsins (comprising OPN1-5)^35, 36^ and that the opsins are linked to TRP channels. OPN4 (known as melanopsin) has been shown to be involved in photoentraiment or regulation of the circadian rhythm^37^. TRP channels have been called “a missing bond in the photoentrainment mechanism of peripheral clocks throughout evolution”^26^.

Our results with red and NIR light are in broad agreement with previously published data from many other investigators^9^. It has been shown that NIR light could produce a biphasic increase in ATP, and a biphasic increase in MMP with a peak response at 3 J/cm^2^ in cultured cortical neurons^38^. One important mechanistic issue in this regard, is the realization that ROS can be produced in mitochondria when the MMP is lowered, but also ROS are produced when the MMP is increased above the baseline. If the MMP is lowered by mitochondrial dysfunction, then the action of PBM in raising the MMP will lead to a drop in the levels of ROS generated in the mitochondria^39^. On the other hand if the mitochondria are normal then the action of PBM will be to raise MMP above baseline, which will lead to a brief and relatively modest burst of ROS^40^.

We were able to distinguish some subtle differences between the effects of blue light and green light, although overall they were broadly similar, especially when compared to the stark differences produced by red and NIR light. While the effects of blue and green to inhibit proliferation of hASCs and to reduce ATP levels, were almost identical, it was clear that blue light produced higher quantities of ROS and a more pronounced drop in MMP compared to green light. On the other hand green light produced a bigger change in intracellular calcium than did blue light, and a slightly more pronounced drop in intracellular pH. This could be explained if blue light had a more damaging effect against mitochondrial chromophores (CCO and other cytochromes) than did green light, as might be expected by the fact that heme prosthetic groups have a much higher absorption in the blue than the green. The more pronounced action of green light in elevating intracellular calcium concentrations could be explained by the action of green light to trigger opening of TRP ion channels by absorption of photons by a member of the opsin family. By contrast, it was striking how similar the responses of the hASCs to red light and NIR light actually were. This similarity underlines the often proposed theory that both wavelength ranges are absorbed by CCO and stimulate mitochondrial respiration, increase ATP, and only produce modest levels of ROS. It was interesting that the CPZ ion channel inhibitor, partly (but not completely) blocked the generation of ROS by green light, and to a lesser extent by blue light. This implies that blue light generates ROS by for instance (an adverse effect in mitochondria) and also by triggering TRPV channel opening. On the other hand, green light produced ROS more by activating TRPV ion channels and less by an adverse effect in mitochondria. It should be noted that the TRPV1 agonist has been shown to signal by (among other pathways) the generation of ROS^41^.

We believe that the most likely explanation for these observations is that there are (at least two) two mechanisms and two different chromophores at play here. The effects of red and NIR light are consistent with a mitochondrial chromophore (giving more ATP, increasing MMP, and producing only a low level of ROS). The different effects of blue and green light may also be mediated by two different chromophores, one for green and another for blue. However there also clearly is considerable overlap between these two wavelength ranges, which might be expected considering that the bandwidth of biological chromophores can be quite wide. For blue light the effects seem to be more likely to adversely affect mitochondria and cause ROS production, while for green light the effects appear to more concern ion channel activation and reduction in intracellular pH.

Evidence is emerging concerning the role of light and heat-activated ion channels in various PBM experiments. There is evidence that green light in particular, can activate TRPV1. Gu *et al*.^30^ studied activation of the TRPV1 channel that had been exogenously expressed in Xenopus oocytes by red (637 nm) and green (532 nm) laser light. They found that green laser produced more activation TRPV1 than red laser as measured by patch-clamp experiments. TRPV channels are involved in mast cell activation leading to degranulation^42^. Laser activation of TRPV1 in mast cells was abrogated by SKF and ruthenium red (broad-spectrum inhibitors of mammalian ion channels). Wu *et al*.^32^ compared histamine release from mast cells with different wavelengths, and found that blue laser was better than red and green had little effect. Yang *et al*.^33^ found that the mast cell degranulation and intracellular calcium increased equally after irradiation with blue or green laser.

We recently published^43^ another study, which examined whether there was a difference in the response of these identical hASCs to two different wavelengths of near-infrared light. We used a 810 nm laser and a 980 nm laser. Interestingly both wavelengths displayed a biphasic dose response with the cells being responsive to much lower energy densities from a 980 nm laser (0.3 J/cm^2^ maximum response), than they did to 810 nm laser (3 J/cm^2^ maximum response). Again we were surprised to discover that the mechanisms of action showed distinct and remarkable differences. Similar to the data reported in the present study, we found that 810 increased MMP and ATP. However 980 nm appeared to activate TRP ion channels, as shown by a pronounced increase in intracellular calcium, and the ability of ion channel blockers (CPZ and SKF) to inhibit the hASC responses.

Our studies on PBM of hASCs taken together (present work and^13, 43^, have highlighted some interesting facts concerning the relative abilities of PBM to stimulate proliferation or to trigger differentiation in stem cells. Admittedly the culture medium is the most important single factor in deciding whether the hASC will proliferate or alternatively differentiate. Various additional factors added to different kinds of medium will specify the exact differentiation pathway that the cells progress down, i.e. to form osteoblasts, neurons, chondrocytes, adipocytes, hepatocytes, etc. Our particular focus was on differentiation into osteoblasts. We found that PBM wavelengths (415 nm/540 nm/980 nm) that activated TRPV ion channels, increased intracellular calcium, reduced ATP, and produced larger amounts of ROS, and all tended to encourage osteogenic differentiation, while at the same time inhibiting proliferation (415 nm/540 nm). On the other hand, wavelengths (660 nm/810 nm) that increased ATP, raised MMP, and only produced modest amounts of ROS, stimulated proliferation, while having no particular effect on differentiation. There are some studies that suggest that treatments that cause some degree of cell stress, can promote hASC differentiation. These treatments may include ROS^44^, heat shock^45^ and gamma irradiation^46^.

In conclusion, blue and green light inhibits proliferation of hASCs, while red and NIR (810 nm) light stimulate proliferation all at the same dose (3 J/cm2). Considering the pronounced biphasic nature of the dose response curve in PBM, it would be necessary to test other doses of light at different wavelengths before drawing any firm conclusions, especially considering the differences we found in the maximum dose between 810 nm and 980 nm^43^. We found differences between the mechanisms of action of blue/green as compared to red/NIR, and even subtle differences between the mechanisms of blue and green. To answer those who point out that blue light does not penetrate tissue, we might point out that a blue light LED device (Philips BlueTouch) has recently received regulatory approval for relief of back pain, suggesting that the clinical application of blue light for PBM applications may not be completely impracticable.
